# The Influence of KE and EW Dipeptides in the Composition of the Thymalin Drug on Gene Expression and Protein Synthesis Involved in the Pathogenesis of COVID-19

**DOI:** 10.3390/ijms241713377

**Published:** 2023-08-29

**Authors:** Natalia Linkova, Vladimir Khavinson, Anastasiia Diatlova, Michael Petukhov, Elizaveta Vladimirova, Maria Sukhareva, Anastasiia Ilina

**Affiliations:** 1 Saint Petersburg Institute of Bioregulation and Gerontology, 197110 Saint Petersburg, Russia; 2 Saint Petersburg Research Institute of Phthisiopulmonology, 191036 Saint Petersburg, Russia; 3 Pavlov Institute of Physiology of Russian Academy of Sciences, 199034 Saint Petersburg, Russia; 4 Petersburg Nuclear Physics Institute Named after B.P. Konstantinov, NRC “Kurchatov Institute”, 188300 Gatchina, Russia; 5 FSBSI Institute of Experimental Medicine, 197022 Saint Petersburg, Russia

**Keywords:** Thymalin, EW dipeptide, KE dipeptide, COVID-19, cytokine storm

## Abstract

Thymalin is an immunomodulatory drug containing a polypeptide extract of thymus that has demonstrated efficacy in the therapy of acute respiratory distress syndrome and chronic obstructive pulmonary disease, as well as in complex therapy related to severe COVID-19 in middle-aged and elderly patients.. KE and EW dipeptides are active substances of Thymalin. There is evidence that KE stimulates cellular immunity and nonspecific resistance in organisms, exerting an activating effect on macrophages, blood lymphocytes, thymocytes, and neutrophils, while EW reduces angiotensin-induced vasoconstriction and preserves endothelium-dependent vascular relaxation by inhibiting ACE2, the target protein of SARS-CoV-2. However, the mechanism of the immunomodulatory action of Thymalin, KE, and EW during COVID-19 remains unclear. To identify the potential mechanism of action underlying the immunomodulatory activity of Thymalin and its active components, EW and KE dipeptides, we assessed inflammatory response in the context of COVID-19. Interactions between EW and KE dipeptides and double-stranded DNA (dsDNA) were investigated by molecular modeling and docking using ICM-Pro. Analysis of the possible effect of EW and KE dipeptides on gene expression and protein synthesis involved in the pathogenesis of COVID-19 was conducted through the use of bioinformatics methods, including a search for promoter sequences in the Eukaryotic Promoter Database, the determination of genes associated with the development of COVID-19 using the PathCards database of human biological pathways (pathway unification database), identification of the relationship between proteins through cluster analysis in the STRING database (‘Search Tool for Retrieval of Interacting Genes/Proteins’), and assessment of the functional enrichment of protein–protein interaction (PPI) using the terms of gene ontology (GO) and the Markov cluster algorithm (MCL). After that, in vitro studying of a lipopolysaccharide (LPS)-induced model of inflammation using human peripheral blood mononuclear cells was performed. ELISA was applied to assess the level of cytokines (IL-1β, IL-6, TNFα) in the supernatant of cells with or without the impact of EW and KE peptides. Blood samples were obtained from four donors; for each cytokine, ELISA was performed 2–4 times, with two parallel experimental or control samples for each experiment (experiments to assess the effects of peptides on LPS-stimulated cells were repeated four times, while additional experiments with unstimulated cells were performed two times). Using molecular docking, GGAG was found to be the best dsDNA sequence in the classical B-form for binding the EW dipeptide, while GCGC is the preferred dsDNA sequence in the curved nucleosomal form for the KE dipeptide. Cluster analysis revealed that potential target genes for the EW and KE peptides encode the AKT1 and AKT2 proteins involved in the development of the cytokine storm. The specific targets for the EW peptide are the *ACE2* and *CYSLTR1* genes, and specific target for the KE peptide is the *CHUK* gene. Protein products of the *ACE2*, *CYSLTR1*, and *CHUK* genes are functionally associated with IL-1β, IL-6, TNF-α, IL-4, and IL-10 cytokines. An in vitro model of an inflammatory reaction demonstrated that Thymalin and EW and KE dipeptides reduced the synthesis of IL-1β, IL-6, and TNF-α cytokines in human peripheral blood mononuclear cells by 1.4–6.0 times. The immunomodulatory effect of Thymalin under the inflammatory response conditions in COVID-19 is based on the potential ability of its active components, EW and KE dipeptides, to regulate protein synthesis involved in the development of the cytokine storm.

## 1. Introduction

According to the World Health Organization, about seven million people have died worldwide over the past three years from complications caused by COVID-19 [1]. Many patients with mild COVID-19 have experienced post-COVID syndrome. This condition comprises a complex of dysfunctions of various systems, including the cardiovascular, respiratory, hormonal, and nervous systems. The most common symptoms of post-COVID syndrome are shortness of breath, increased blood pressure, impaired thyroid function, tachycardia, and arrhythmia [2,3]. Thus, even taking into account the cessation of pandemic status, COVID-19 remains a topical medical and social issue. The RNA-containing virus SARS-CoV-2 uses the ACE2 receptor to enter the cell and further replicate in it. Other proteins, such as TMPRSS2 and cathepsin L, are also involved in the penetration of the virus into the cell [4]. The extracellular domain of ACE2 is located on lung alveolar epithelial cells, as well as on arterial and venous endothelial cells, arterial smooth muscle cells, renal tubular epithelium, and the small intestine. Suppression of ACE2 expression in tissues in COVID-19 patients leads to dysfunction of the renin–angiotensin system (RAS) and dysregulation of blood pressure [5]. In addition, replication of SARS-CoV-2 in airway epithelial cells can lead to the development of pyroptosis with vascular damage. As a result, local inflammation occurs, which initiates the interaction of macrophages with foreign agents and the secretion of pro-inflammatory cytokines and chemokines, such as IL-1, IL-6, IL-8, IL-21, TNF-β, and MCP-1. Cytokines promote the migration of lymphocytes and leukocytes to the site of infection, resulting in a “cytokine storm”—an acute inflammatory reaction of the immune system in response to infection. Cytokine storms can be accompanied by dysfunctions of organs that may result in death [6].

At an early stage of the disease, the activation of monocytes and macrophages producing IL-1β, IL-2, IL-6, and TNFα is observed [7]. Maximum concentrations of cytokines IL-1β, IL-2, IL-6, IL-7, IL-8, IL-10, G-CSF, GM-CSF, and MCP-1 are observed in the blood of patients with severe COVID-19 on day 8–11. In mild and moderate forms of the disease, the concentrations of pro-inflammatory cytokines in the blood are also increased, but to a lesser extent than in the severe form of COVID-19 [7,8].

Thymalin is an immunomodulatory drug containing a polypeptide extract of thymus. The efficacy of Thymalin has been demonstrated in the therapy of acute respiratory distress syndrome and chronic obstructive pulmonary disease, as well as in complex therapy related to severe COVID-19 in middle-aged and elderly patients [9]. It has been established that Thymalin contributes to normalization of the number of lymphocytes, monocytes, leukocytes, NK cells, blood platelets, and indicators of the hemostasis system (fibrinogen, lactate dehydrogenase, D-dimer) during coronavirus infection. The efficacy of Thymalin in severe COVID-19 is superior to that of Tocilizumab, which is supported by data on reduced patient mortality rates [9,10].

Thymalin has been shown to reduce the expression of CD44 (stem cells marker) and CD117 (marker of the intermediate stage of the differentiation of hematopoietic stem cells, HSCs) and increase the expression of CD28 (marker of mature T-lymphocytes), which indirectly indicates that Thymalin stimulates the differentiation of CD117+ cells into mature CD28+ T lymphocytes [11]. It has been previously shown that in patients with severe COVID-19, the number of blood CD28+, CD4+, and CD8+ T-lymphocytes decreases, which is indicative of pronounced immunity suppression [12]. This suggests that the antiviral effect of Thymalin involves compensatory stimulation of HSC differentiation into CD28+ T-lymphocytes at the stage of immunosuppression during the unfavorable course of a viral infection.

The short immunoprotective peptides KE and EW are the components of Thymalin [5]. The KE peptide stimulates cellular immunity and the nonspecific resistance of organisms, exerting an activating effect on macrophages, blood lymphocytes, thymocytes, and neutrophils. This dipeptide induces the expression of CD4 and CD5 molecules on thymic cells, stimulating their differentiation towards T-helpers. Short tryptophan-containing peptides (EW in particular) have been demonstrated to reduce angiotensin-induced vasoconstriction and preserve endothelium-dependent vascular relaxation [13] by inhibiting ACE2, the target protein of SARS-CoV-2, which makes them promising candidates for investigation in the complex therapy of COVID-19 and post-COVID syndrome. It has been shown that short peptides, including KE and EW, can penetrate into the nucleus and nucleolus of cells and interact with the nucleosome, histone proteins, and single- and double-stranded DNA, which suggests peptide regulation of gene expression, protein synthesis, and chromatin state [5].

The ability of KE and EW dipeptides to bind with dsDNA leads us to think that dipeptides, and, probably, Thymalin, could regulate the expressions of genes that are associated with COVID-19. Thus, one of the objectives of our research was to find the specific sites where KE and EW peptides bind with dsDNA using molecular dynamic methods and then find the appropriate gene promotors associated with COVID-19 and include those sites using bioinformatic methods.

Taking into account the ability of Thymalin and KE and EW dipeptides to modulate immunity, on the one hand, and regulate genes expression, on the other, we have assumed that the potential mechanism of their action could include the regulation of proinflammatory cytokine production, such as IL-1β, IL-6, and TNFα. Therefore, the other objective of the paper was to investigate the efficacy of the Thymalin and EW and KE dipeptides in reducing proinflammatory cytokine production in vitro using a lipopolysaccharide (LPS)-induced inflammatory model.

## 2. Results

### 2.1. Identification of the Binding Sites of EW and KE Dipeptides to the Classical B-Form of Double-Stranded DNA and the Nucleosome

The possibility of forming complexes of the EW peptide with dsDNA sites GGAG (ICM-Score = −32.6 kcal/mol) and AGAC (ICM-Score = −32.1 kcal/mol) was demonstrated. When interacting with the GGAG dsDNA site, the EW peptide forms four intermolecular hydrogen bonds on the side of the minor groove with nitrogen atoms at the guanine G5, G6, and adenine A7 positions. When interacting with the AGAC dsDNA site, the EW peptide forms four intermolecular hydrogen bonds on the side of the minor groove with nitrogen atoms at the guanine G6 and adenine A7 positions and a phosphate backbone at the thymine T20 and T21 positions.

The positions of the EW dipeptide in complexes with GGAG and AGAC coincide. In the central part of dsDNA, guanine G6 forms a hydrogen bond with the carbonyl group of the main peptide chain, while adenine A7 forms a hydrogen bond with the nitrogen atom of the C-terminal tryptophan. The replacement of guanine G5 with adenine A5 results in the loss of the hydrogen bond donor for the N-terminal glutamic acid and, consequently, the loss of the hydrogen bond energy contribution. However, in the case of interaction with the AGAC site, the value of the Van der Waals interaction energy decreased (from −25 kcal/mol to −30 kcal/mol), while the polarization of chemical bonds increased, which resulted in a positive contribution to the interaction energy.

The best ICM-Score for the interaction of the KE peptide with the curved dsDNA region GCGC (nucleosome model) was −30.7. However, results obtained earlier on another dsDNA model showed the possibility of the formation of a stable complex (ICM-Score function value ≤ −32) of the KE dipeptide with the TCGA dsDNA region in the classical B-form, with the ICM-Score function value = −35.8 [14]. When interacting with the TCGA dsDNA region, the KE dipeptide forms four intramolecular and six intermolecular hydrogen bonds on the minor groove side: the N-terminal peptide group with the cytosine carbonyl group at the C6 position; N-terminal lysine with the thymine carbonyl group T5, the adenine nitrogen atom A21, and a phosphate backbone oxygen at the A22 position; and the carboxyl group of C-terminal glutamic acid with the guanidine amino group at positions G7 and G19.

### 2.2. Identification of COVID-19-Associated Target Genes for EW and KE Peptides

**Figure 1:**
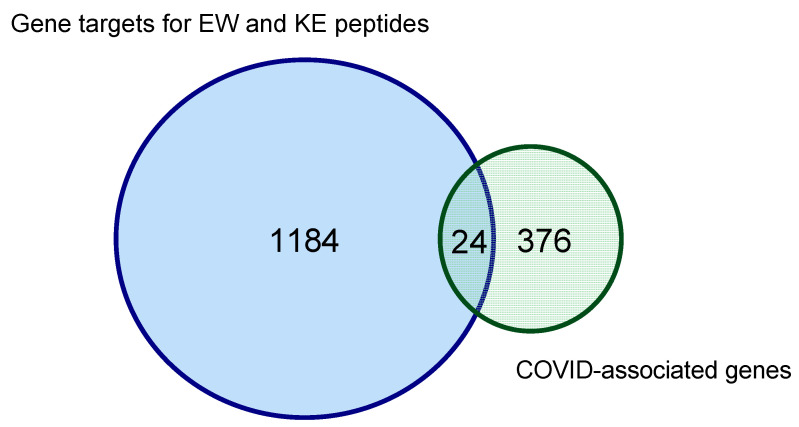
Euler diagram showing the ratio of human genes potentially under the control of short peptides and genes associated with the development of COVID-19 according to search results in the EPD database. The intersection of the circles indicates the number of genes associated with the development of COVID-19, which are potential targets for KE and EW peptides.

Using the EPD database, which contains 29,598 promoters of human genes, the GGAG dsDNA sequence was found in 542 promoters. This suggests that the EW peptide can regulate the expression of 542 human genes. The GCGC dsDNA sequence, which forms the most energetically favorable complex when combined with the KE peptide, was found in 642 promoters of human genes. Thus, the KE peptide potentially regulates the expression of 642 human genes. Therefore, 1184 out of 29,598 human genes are potential targets for KE and EW peptide binding (Figure 1).

**Figure 2:**
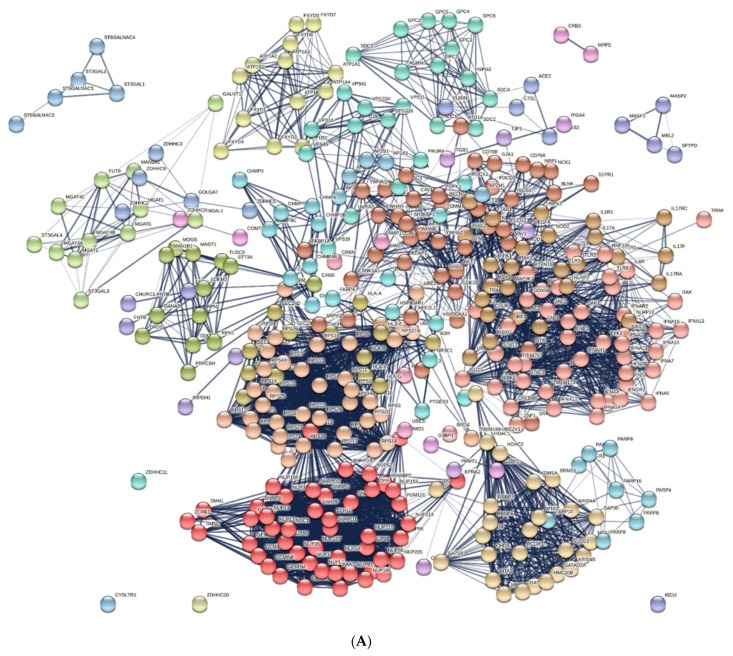

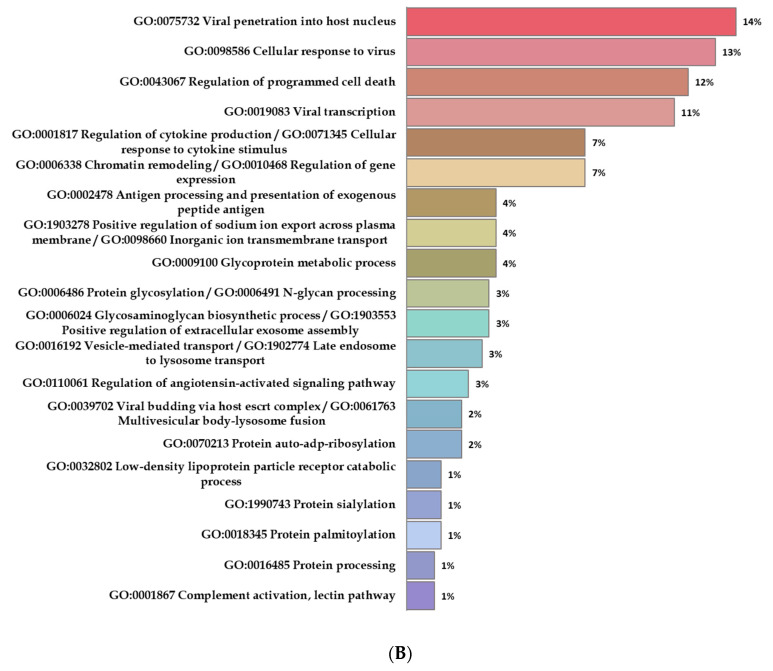
The set of proteins encoded by COVID-19-associated genes according to the PathCards database. (**A**) represents the network of protein–protein interactions, and (**B**) represents the proportion of proteins in biological processes according to GO terms.

The PathCards database identified 376 genes involved in the pathogenesis of COVID-19. A total of 354 genes are included in 20 clusters that had gene ontology (GO) terms associated with viral penetration into the host nucleus, cellular response to the virus, viral transcription, the regulation of cytokine production, viral budding via host ESCRT complex, etc. (Figure 2).

Proteins encoded by the *CHURC1-FNTB*, *FNTA*, *FNTB*, *IMPDH1*, *IMPDH2*, *TJP1*, *VEGFA*, *CNBP*, *PRMT1*, *ITGA4*, *ITGB1*, *G3BP1*, *G3BP2*, *COMT*, *ZDHHC8*, *CRB3*, *MPP5*, *UBE2I*, *MAP1LC3B*, *RBX1*, *CRBN*, and *BRD4* genes formed clusters of less than four elements and were not considered in terms of functional relationships.

Of all 376 genes associated with the development of COVID-19, genes whose promoters contained nucleotide sequences for the specific binding of EW and KE peptides were identified. Fifteen promoters of genes (*AKT2*, *CHMP4B*, *CHMP4A*, *ACE2*, *CYSLTR1*, *CUL3*, *DDX20*, *DAD1*, *CHMP3*, *CHMP7*, *DDX5*, *CHMP4C*, *AKT1*, *AGRN*, *CHMP6*) involved in the development of COVID-19 were found for the EW peptide according to the PathCard database, and nine promotors of COVID-associated genes were found for the KE peptide (*AKT2*, *CHMP4B*, *CSNK1A1*, *CUL3*, *DAD1*, *CHMP2B*, *CHUK*, *AKT1*, *AGRN*). At the same time, some specific genes were identified: CHMP4A, ACE2, YSLTR1, DDX20, CHMP3, CHMP7, DDX5, CHMP4C, and CHMP6 for the EW peptide and CSNK1A1, CHMP2B, and CHUK for the KE peptide. Thus, among the COVID-19-associated genes, there were 24 identified genes (6.4%), the promoters of which are potential targets for the binding of EW and KE peptides.

Based on the results of the in vivo COVID-19 cytokine storm study [7,15,16,17], we focused on the IL-1β, IL-2, IL-6, TNFα, IL-4, and IL-10 molecular pathways as the key elements of the inflammatory response in order to identify the molecular mechanisms of peptidergic regulation of the cytokine storm in COVID-19. We studied the relationship of proteins encoded by the COVID-associated target genes of EW and KE peptides with cytokines, whose gene expression was studied in vitro.

**Figure 3:**
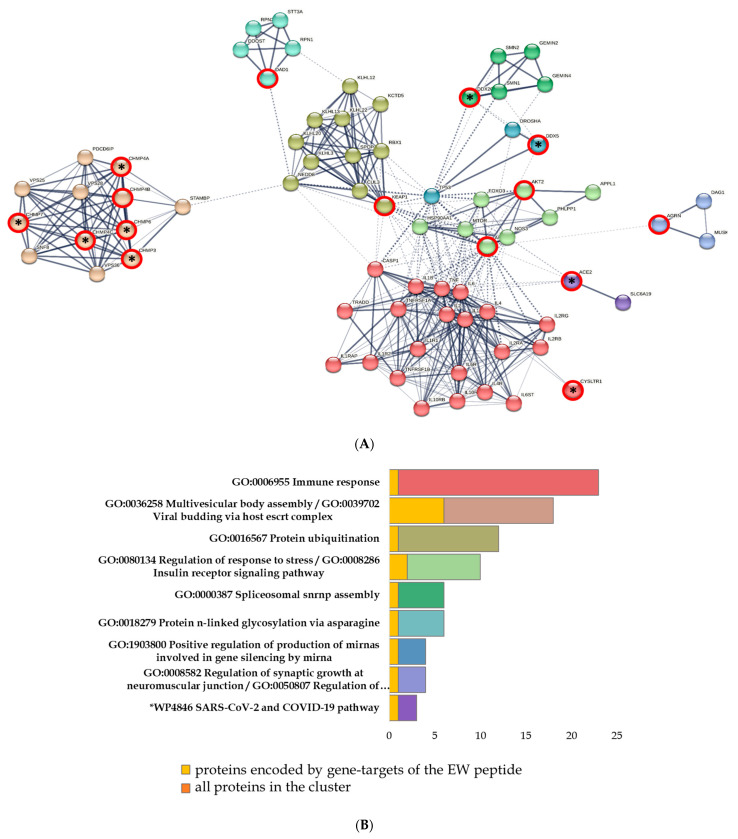
Functional analysis of the interactions between cytokine storm proteins and proteins encoded by EW peptide target genes. Designations: red circles signify proteins encoded by peptide target genes; red circles with an asterisk signify proteins encoded by specific EW peptide target genes. (**A**) Saturated network of protein–protein interactions; (**B**) the proportion of proteins in biological processes.

When analyzing the network of protein–protein interactions formed by the saturation of the set of proteins encoded by the COVID-associated EW peptide target genes and cytokine storm proteins, nine clusters were identified according to GO terms. In these clusters, proteins have been associated with various biological processes, such as immune response (CASP1, CYSLTR1, IL10, IL10RA, IL10RB, IL1B, IL1R1, IL1R2, IL1RAP, IL2, IL2RA, IL2RB, IL2RG, IL4, IL4R, IL6, IL6R, IL6ST, TNF, TNFRSF1A, TNFRSF1B, TRADD), multivesicular body assembly/viral budding via host ESCRT complex (CHMP3, CHMP4A, CHMP4B, CHMP4C, CHMP6, CHMP7, PDCD6IP, SNF8, STAMBP, VPS25, VPS28, VPS36), protein ubiquitination (CUL3, KCTD5, KEAP1, KLHL12, KLHL13, KLHL20, KLHL22, KLHL3, NEDD8, RBX1, SPOP), regulation of immune system and apoptotic processes (AKT1, AKT2, APPL1, FOXO3, HSP90AA1, MTOR, NOS3, PHLPP1), spliceosomal SNRNP assembly (DDX20, GEMIN2, GEMIN4, SMN1, SMN2), protein n-linked glycosylation via asparagine (DAD1, DDOST, RPN1, RPN2, STT3A), positive regulation of the production of miRNAs (DDX5, DROSHA, TP53), regulation of synaptic growth at the neuromuscular junction (AGRN, DAG1, MUSK), and the WP4846 SARS-CoV-2 and COVID-19 pathway (ACE2, SLC6A19) (Figure 3).

Upon further investigation of the functional network of protein–protein interactions, we identified the links between the cytokine storm proteins and proteins encoded by target genes for EW peptide binding (Table 1). Of the 15 target genes for EW peptide binding, only 4 were capable of encoding proteins, which are functionally associated with the cytokine storm proteins, namely ACE2 c IL1B, IL6, TNFA; AKT1 c IL-2, IL-4, IL-1B, Il-10, IL6, TNFA; AKT2 c IL6, TNFA; and CYSLTR1 c IL4, TNFA.

**Figure 4:**
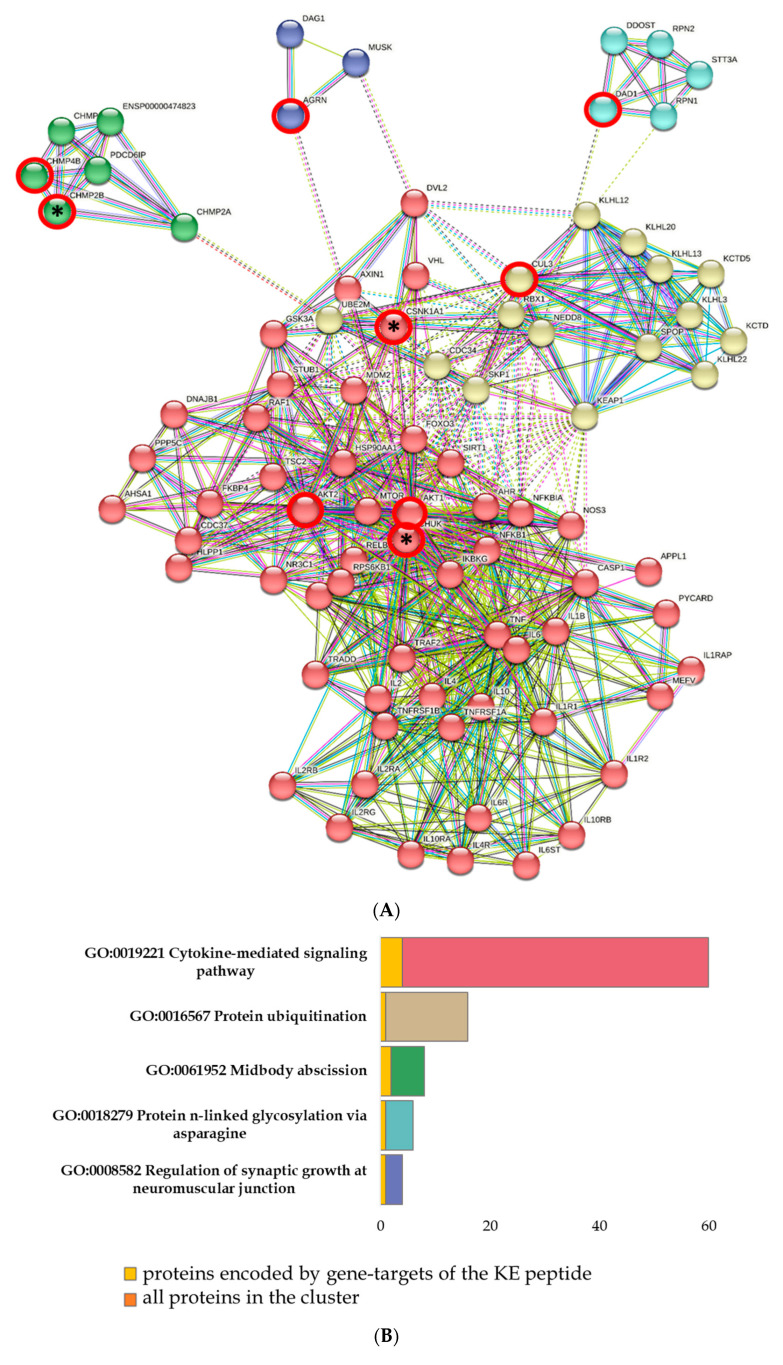
Functional analysis of the interactions between cytokine storm proteins and proteins encoded by KE peptide target genes. Designations: red circles signify proteins encoded by peptide target genes; red circles with an asterisk signify proteins encoded by specific KE peptide target genes. (**A**) Saturated network of protein–protein interactions. (**B**) The proportion of proteins in biological processes.

When analyzing the network of protein–protein interactions formed by the saturation of the set of proteins encoded by the COVID-associated KE peptide target genes and cytokine storm proteins, five clusters were identified according to GO terms. In these clusters, proteins have been associated with various biological processes, such as cytokine-mediated signaling pathways (AKT1, AKT2, APPL1, AXIN1, CASP1, CHUK, CSNK1A1, FOXO3, HSP90AA1, IKBKG, IL10, IL10RA, IL10RB, IL1B, IL1R1, IL1R2, IL1RAP, IL2, IL2RA, IL2RB, IL2RG, IL4, IL4R, IL6, IL6R, IL6ST, MDM2, MTOR, NFKB1, NFKB2, NFKBIA, NOS3, PHLPP1, RELB, TNF, TNFRSF1A, TNFRSF1B, TRADD, TRAF2), protein ubiquitination (CUL3, KCTD10, KCTD5, KEAP1, KLHL12, KLHL13, KLHL20, KLHL22, KLHL3, NEDD8, RBX1, SPOP), midbody abscission (CHMP2A, CHMP2B, CHMP3, CHMP4B, ENSP00000474823, PDCD6IP), protein n-linked glycosylation via asparagine (DAD1, DDOST, RPN1, RPN2, STT3A), and the regulation of synaptic growth at neuromuscular junctions (AGRN, DAG1, MUSK) (Figure 4).

Upon further investigation of the functional network of protein–protein interactions, we identified the closest links between the proteins of the cytokine storm and proteins encoded by target genes for KE peptide binding (Table 2). Of the nine target genes for KE peptide binding, only three encoded proteins that were functionally associated with cytokine storm proteins, namely AKT1 with IL-2, IL-4, IL-1B, IL-10, IL-6, and TNFA; AKT2 with IL-6 and TNFA; and CHUK with IL-10, IL6, and TNFA.

**Figure 5:**
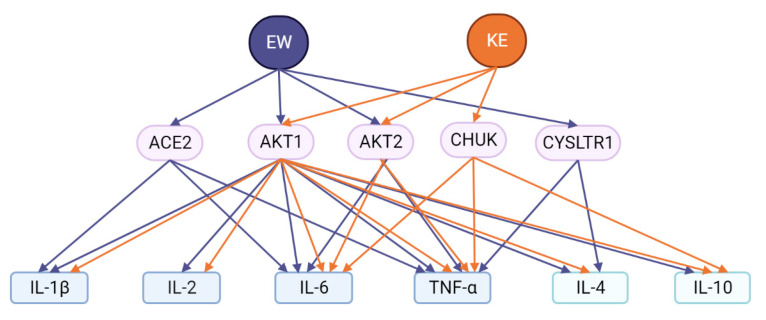
Potential functional relationship of short EW and KE peptides with cytokines.

Thus, out of all 14 proteins encoded by the target genes of the EW and KE peptides, AKT1 and AKT2 proteins are common for both said peptides as potential intermediates in the peptide regulation of the cytokine storm. At the same time, ACE2 and CYSLTR1 proteins are specific for the EW peptide and are functionally associated with IL-1β, IL-6, TNF-α, and IL-4 cytokines. The CHUK protein is an intermediate functional element for the KE peptide and is associated with the IL-6, TNF-α, and IL-10 cytokines (Figure 5).

### 2.3. Thymalin and Dipeptides EW and KE: Effect on Cytokine Release in Blood Mononuclear Cells in an In Vitro Inflammatory Response Model

Taking into account the results of molecular modeling and cluster analysis, which demonstrated the potential of KE and EW dipeptides to bind to dsDNA and regulate cytokine signaling, the effect of KE and EW, as well as the drug Thymalin, on the synthesis and release of a set of pro-inflammatory cytokines (TNF-α, IL-1β, IL-6) by peripheral blood mononuclear cells was studied. The inflammatory response was modeled by adding LPS to cell cultures. The results are provided in Table 3. Pilot experiments were also carried out to investigate the peptides’ effects on PBMCs without LPS stimulation, that is, the ability of the peptides themselves to modulate the functional activity of these cells was evaluated.

It was found that when peptides were incubated at a concentration of 1 mg/mL with human PBMCs stimulated by LPS, a statistically significant decrease in the release of the pro-inflammatory cytokine TNF-α was observed in all cases (by 6 times for the KE peptide, by 5 times for the EW peptide, and by 2.2 times for Thymalin). Interestingly, the KE peptide suppressed the release of TNF-α by untreated PBMCs by 4.9 times, while the EW peptide suppressed release by 11.4 times and Thymalin did not have a significant effect on TNF-α synthesis by control cells. At the same time, Thymalin, in contrast to the KE and EW peptides, statistically significantly reduced the synthesis of IL-1β in PBMCs stimulated by LPS by 2.7 times. Thymalin and EW had no effect on IL-1β synthesis by non-LPS-treated PBMCs, while KE suppressed the release of this cytokine by 2.1 times. The KE peptide reduced the synthesis of IL-6 by PBMCs stimulated by LPS by 2.5 times. Peptides KE and EW reduced the synthesis of IL-6 by non-LPS-treated PBMCs by about two times, while Thymalin, on the contrary, stimulated the release of IL-6 by non-LPS-treated PBMCs by about four times.

## 3. Discussion

For the dipeptides EW and KE, the possibilities of binding to dsDNA regions in the classical B-form and in the twisted form of four residues were analyzed using molecular modeling methods and the computer docking of ligands.

As a result, the DNA nucleotide sequences and the corresponding conformations of dsDNA complexes with peptides were determined, which, according to the calculation of the ICM-Score function, had the highest affinity for dsDNA. The minimum value of the ICM-Score function for different peptides was found among dsDNA regions both in the classical B-form and in the curved one. It was found that EW and KE peptides in the obtained complexes were located on the side of the minor groove. The results obtained indicate that the efficiency of peptide binding to dsDNA is significantly affected by the dsDNA sequence and the curvature of the dsDNA helix. Thus, it is necessary to consider not only unique sites but also different conformations of dsDNA when studying the interaction of peptides with dsDNA.

Taking into account the data on the binding sites of short peptides with dsDNA obtained at the stage of molecular modeling, a cluster analysis was carried out to identify potential functional links in the molecular mechanism of peptidergic regulation of the cytokine storm in COVID-19. This analysis identified 24 target genes for short peptide binding associated with COVID-19 development.

Comparative analysis of two peptides has shown that they have common binding sites in the *AKT1* and *AKT2* genes, the protein products of which are functionally associated with the development of the cytokine storm in COVID-19. On the other hand, each of the peptides also has specific binding sites in the *ACE2* and *CYSLTR1* genes (for the EW peptide) and in the *CHUK* gene (for the KE peptide).

The *AKT1* and *AKT2* genes encode members of the human AKT serine–threonine protein kinase family. AKT/PI3K forms a key component of many signaling pathways that involve the binding of membrane-bound ligands such as receptor tyrosine kinases, G-protein coupled receptors, and integrin-linked kinase. These AKT proteins therefore regulate a wide variety of cellular functions, including cell proliferation, survival, metabolism, and angiogenesis in both normal and malignant cells. The PI3K/AKT signaling pathway regulates clathrin-mediated endocytosis, and inhibition of this pathway suppresses viral entry [18].

The *ACE2* gene encodes angiotensin-converting enzyme 2 (ACE2), a highly expressed protein in the upper and lower respiratory tract cells, whose function under physiological conditions is to counteract the renin–angiotensin system and reduce blood pressure by catalyzing the hydrolysis of an AT2 vasoconstrictor peptide into AT1–7 and AT1–9 vasodilators. The SARS-CoV-2 virus enters the host cell through the interaction of its Spike or S protein with the ACE2 receptors of the host cell. Therefore, the ACE2 gene is a critical protein in terms of susceptibility to SARS-CoV-2 infection, acting as its gateway to the host cell. It has been shown that higher expression of the *ACE2* gene is associated with a higher risk of SARS-CoV-2 infection [19].

This *CYSLTR1* gene encodes cysteinyl leukotriene receptor 1 (CYSLTR1), which is a member of the G-protein coupled receptor 1 family and involved in mediating bronchoconstriction. Activation of CYSLTR1 results in contraction and proliferation of bronchial smooth muscle cells, eosinophil migration, and damage to the mucus layer in the lung. Upregulation of this gene is associated with asthma. Dysregulation of CYSLTR1 expression reflects the functional activity of dendritic cells that are involved in the development of an adaptive immune response [20].

The *CHUK* gene encodes the component of a cytokine-activated protein complex that is an inhibitor of the essential transcription factor NF-kappa-B complex (CHUK), subsequently phosphorylating sites that trigger the degradation of the inhibitor via the ubiquination pathway, thereby activating the transcription factor. Serine kinase plays an essential role in the NF-kappa-B signaling pathway, which is activated by multiple stimuli such as inflammatory cytokines, bacterial or viral products, DNA damage, or other cellular stresses. Therefore, CHUK plays a key role in the negative feedback of NF-kappa-B canonical signaling to limit inflammatory gene activation [21].

The presence of common target genes and, accordingly, functional molecules in the mechanism of action of peptides suggests a general principle of peptide regulation of processes developing during COVID-19. At the same time, the identification of specific potential targets for each peptide indicates the possible synergistic nature of their action, which is manifested in clinical practice by the high efficiency of the peptide drug Thymalin, which contains the above peptides.

To verify the obtained data in vitro, we studied the effect of EW and KE short peptides and the drug Thymalin on the synthesis and release of cytokines by human peripheral blood mononuclear cells stimulated with bacterial lipopolysaccharides. It was established that the short peptides EW and KE (which are the components of Thymalin), as well as Thymalin itself, indeed affected the synthesis of cytokines in an inflammation model. The TNF-α cytokine was the most susceptible to the modulatory action of the studied peptides; EW and KE peptides inhibited its release by PBMCs under the influence of LPS almost to the level of intact cells. In addition, the peptides had a modulating effect on the synthesis of TNF-α by non-LPS-treated PBMCs, reducing its level by 4.9–11.4 times. Thymalin had a less pronounced effect on LPS-induced TNF-α synthesis in PBMCs, reducing it two-fold compared to the control. Interestingly, only Thymalin had an inhibitory effect on the synthesis of IL-1β by LPS-stimulated PBMCs, reducing it by 2.7 times. At the same time, Thymalin stimulated IL-6 in non-LPS-treated cells, increasing its value to the level of IL-6 in LPS-stimulated cells.

## 4. Materials and Methods

### 4.1. Molecular Modeling of the EW and KE Dipeptides’ Interaction with Double-Stranded DNA and Nucleosomes

In this paper, we studied the interaction of EW and KE peptides with double-stranded DNA (dsDNA) in the classical B-form, as well as in the curved nucleosomal form, using widely used methods for the molecular docking of flexible ligands. The ICM-Pro software package (MolSoft, San Diego, CA, USA) was used. At the preparatory stage, the spatial structures of the EW and KE peptides were generated using the ICM-Pro molecular editor and its library of the 20 standard amino acids. The library of 136 dsDNA sequences, consisting of 4 base pairs having unique spatial structures, was used as described earlier [14]. Based on the complementarity and symmetry of the spatial structure of dsDNA in the classical B-form, 136 dsDNA sequences, consisting of 4 base pairs having a unique spatial structure, were obtained. Using the ICM language software scripts, spatial structures of DNA receptors in the classical linear B-form were generated, consisting of two repeated pairs of nucleotides (the first and last four pairs were AT-rich regions) and alternating sequences of the site under study [14]. To obtain a curved DNA shape, these structures were minimized in the force field of the ICM-Pro software package (ICMFF) [22] with the imposition of spatial restrictions on their structures until the helix was twisted in accordance with the dsDNA site in the composition of human nucleosomes. The peptide binding site was defined as the central alternating sequences of the dsDNA molecule site under investigation.

Molecular docking of the studied ligands was performed in the force field of the ICM-Pro software package and the DockScan algorithm (Molsoft LLC) [23] using a multiprocessor supercomputer at the Petersburg Nuclear Physics Institute Named after B.P. Konstantinov, the NRC “Kurchatov Institute”. In the calculations, global optimization of the conformational energy of the flexible ligand in the receptor force field (ICMFF) was performed. Using the Monte Carlo method, stochastic conformations of the EW and KE peptides were obtained with further local minimization of the energy gradient [22]. The docking of the peptides was carried out with maximum care (Thorough = 30) and selected experimentally based on the 90% reproducibility of docking results, as described previously [14]. After the virtual screening procedure, the obtained DNA–peptide complexes were sorted to retain complexes with the lowest ICM-Score function values for further analysis.

### 4.2. Bioinformatics

The first stage of the study revealed the affiliation of the KE and EW peptide binding sites, found by molecular modeling, to the promoter regions of genes associated with COVID-19 development. These genes were considered as potential targets for peptide binding. The search for promoter sequences of human genes was carried out in the Eukaryotic Promoter Database (EPD) (database URL: http://www.epd.isb-sib.ch accessed by the 1 June 2023) [24]. Nucleotide sequences identified as the best binding sites to DNA for the EW (GGAG) and KE (GCGC) peptides were implemented as a search query. Genes associated with the development of COVID-19 were determined using the PathCards database of human biological pathways (pathway unification database) (database URL: http://pathcards.genecards.org/ (accessed on 1 June 2023)) [25]. The search query consisted of “SARS-CoV” followed by “SARS-CoV-2 Infection”. During the next stage, a functional analysis of the relationship between the proteins encoded by COVID-19-associated genes and KE and EW peptide binding targets was performed. To identify the relationship between proteins, cluster analysis was carried out in the STRING database (‘Search Tool for Retrieval of Interacting Genes/Proteins’, 11.0) (database URL: https://string-db.org/ (accessed on 1 June 2023)) [26]. The relationship of proteins associated with the development of COVID-19 was visualized using the following basic parameters: (i) a full STRING network; (ii) active interaction sources (text mining, neighborhood, experiments, gene fusion, databases, co-occurrence, co-expression); (iii) medium confidence (0.4); and (iiii) no interactors on the first and the second shells. To visualize the network of proteins encoded by target genes for short peptides, the network was saturated under the condition of 50 interactors on the first shell and none on the second shell [27]. To assess the functional enrichment of protein–protein interactions (PPIs) using the terms of gene ontology (GO), the Markov cluster algorithm (MCL) was used with the following parameters: inflation parameter—3, minimum required interaction score—0.4. The PPI enrichment *p*-value was <0.05. The assignment of GO terms to clusters was carried out depending on the following indicators: “count in network”, “strength”, or “false discovery rate”. Clusters consisting of more than 4 proteins were considered. For quantitative analysis and plotting, Excel 2016, GraphPad Software 9.5.1.733, and BioRender tools were used.

### 4.3. Isolation of Human Peripheral Blood Mononuclear Cells

We used blood samples obtained from 4 donors, who were healthy men and women of middle-age. Phosphate buffered saline (PBS) was added to 15–20 mL of heparinized peripheral blood containing 500 µL of heparin in a ratio of 1:1. The contents of the tube were layered on a Ficoll-400 solution with a density of 1.077 (Pharmacia, Stockholm, Sweden) in a volume of 12–15 mL. Mononuclear cells were isolated by centrifugation at 600× *g* for 40 min at 4 °C. The resulting cells were washed twice in 20 mL of PBS by centrifugation at 600× *g* for 10 min, then resuspended in 3–5 mL of RPMI-1640 nutrient medium (Biolot, Saint Petersburg, Russia); the number of cells was counted in a Goryaev chamber. The procedure for obtaining mononuclear cells from donor blood was reviewed and approved by the local ethics committee at the FSBSI “Institute of Experimental Medicine”, St. Petersburg, Russian Federation (protocol 1/20 from 27 February 2020). All blood donors gave written informed consent.

### 4.4. Cell Culture, Treatments, and ELISA

Thymalin and the short peptides KE and EW (1 mg/mL) were added to mononuclear cells at a concentration of 4 × 10^6^ cells/mL and incubated in 96-well plates for 18 h in a CO_2_ incubator (5% CO_2_) at 37 °C. The production and release of cytokines in cells was stimulated by the introduction of bacterial LPS (lipopolysaccharides from *Escherichia coli* O55:B5, L2880-25MG, Sigma, St. Louis, MO, USA), which is a generally recognized and widely used model of inflammation induction in blood mononuclear cell cultures [28]. After the incubation, the plates with cells were centrifuged for 5 min at 1000 rpm. The level of cytokines (IL-1β, IL-6, TNFα) in the supernatant was determined using kits for an enzyme-linked immunosorbent assay (ELISA) (OOO Cytokin, Saint Petersburg, Russia; Vector-Best, Novosibirsk, Russia). The optical density of the samples was measured at a wavelength of 450 nm on a SpectraMax250 microplate spectrophotometer (Molecular Devices, San Jose, CA, USA), and the concentration of the studied cytokines was calculated. Experimental data from the spectrophotometer were obtained using the SoftMaxPro5 program. Since the duration of incubation was relatively long (18 h), we suppose that the value of the cytokine level reflected both the intensity of their release from cells and the stimulation/inhibition of their synthesis.

For each cytokine, ELISA was performed 2–4 times, with two parallel experimental or control samples for each experiment (experiments to assess the effects of peptides on LPS-stimulated cells were repeated 4 times, while additional experiments with unstimulated cells were mainly repeated 2 times). The concentration of cytokines was expressed as % of the control (LPS), i.e., the cytokine concentration in samples where LPS was added in the absence of a peptide. The results were calculated as medians based on data from 2–4 independent experiments. To identify statistically significant differences between the parameters in the experimental and control groups, the non-parametric Mann–Whitney test was used to compare two independent groups in the StatSoft STATISTICA 10.0.1011 program.

## 5. Conclusions

Taking into account the published data concerning COVID-19 process correction using Thymalin [9,29,30], our investigation aimed to reveal new aspects of the immunomodulatory action of Thymalin and its active components, dipeptides EW and KE.

Molecular modeling data on the binding of EW and KE peptides with dsDNA and nucleosomes, as well as the results of cluster analysis, allowed us to identify 24 target genes of peptide regulation associated with COVID-19. In addition, in vitro study using an LPS-induced model of inflammation allowed us to obtain new results regarding the action of Thymalin and EW and KE peptides on proinflammatory cytokine (IL-1β, IL-6, TNFα) production. Further investigation of the immunomodulatory effects of Thymalin and EW and KE dipeptides should involve the study of their action in terms of COVID-19-associated gene expression, as well as protein synthesis. Future research is promising in terms of identifying the best methods of application for these peptides in the treatment of COVID-19 and correction of cytokine storm-induced disorders.

## Figures and Tables

**Table 1:** Functional links between proteins encoded by EW peptide target genes and proteins involved in the cytokine storm.

No.	Proteins Encoded by EW Peptide Target Genes	Proteins Involved in the Cytokine Storm	Evidence Suggesting Functional Links
1.	ACE2 *	IL1B, IL6, TNFA	Co-Mentioned in Pubmed Abstracts PMID:32222466, PMID:3226989, PMID:32635752, PMID:32121598, PMID:31746626, PMID:32247927
2.	AGRN	–	
3.	AKT1	IL-2, IL-4, IL-1B, Il-10, IL6, TNFA	Co-Mentioned in Pubmed Abstracts PMID:31918290, PMID:32251678, PMID:32117985, PMID:32132672, PMID:32059381
4.	AKT2	IL6, TNFA	Co-Mentioned in Pubmed Abstracts PMID:32059381, PMID:35016612
5.	CHMP3 *	–	
6.	CHMP4A *	–	
7.	CHMP4B	–	
8.	CHMP4C *	–	
9.	CHMP6 *	–	
10.	CHMP7 *	–	
11.	CUL3	–	
12.	CYSLTR1 *	IL4, TNFA	Co-Mentioned in Pubmed Abstracts PMID:31936183, PMID:30516547, PMID:31592409, PMID:31231429, PMID:31781316
13.	DAD1	–	
14.	DDX20 *	–	
15.	DDX5 *	–	

Note: *—protein is encoded by a target gene specific to the EW peptide.

**Table 2:** Functional links between proteins encoded by KE peptide target genes and proteins involved in the cytokine storm.

No.	Proteins Encoded by KE Peptide Target Genes	Proteins Involved in the Cytokine Storm	Evidence Suggesting Functional Links
1.	AKT1	IL-2, IL-4, IL-1B, Il-10, IL6, TNFA	Co-Mentioned in Pubmed Abstracts PMID:31918290, PMID:32251678, PMID:32117985, PMID:32132672, PMID:32059381
2.	AKT2	IL6, TNFA	Co-Mentioned in Pubmed Abstracts PMID:32059381, PMID:35016612
3.	CHMP2B *	–	
4.	CHMP4B	–	
5.	CHUK *	Il-10, IL6, TNFA	Co-Mentioned in Pubmed Abstracts PMID:31988590, PMID:32442901
6.	CSNK1A1 *	–	
7.	CUL3	–	
8.	DAD1	–	
9.	AGRN	–	

Note: *—the protein is encoded by a target gene specific to the KE peptide.

**Table 3:** Effect of short peptides on the release of cytokines (TNF-α, IL-1β, IL-6) by LPS-stimulated human peripheral blood mononuclear cells (PBMCs) and unstimulated cells.

Peptides, 1 mg/mL	The Concentration of Cytokines in % of the “LPS” Control					
	TNF-α		IL-1β		IL-6	
	With LPS	No LPS	With LPS	No LPS	With LPS	No LPS
KE	16.8 *	**3.5 ^#^**	56.9	5.4	39.4 *	**10.9 ^#^**
EW	19.7 *	**1.5 ^#^**	70.9	12.3	47.5	**11.4 ^#^**
Thymalin	44.9 *	10.3	37.5 *	12.3	80.0	**79.9 ^#^**
Control (no LPS or peptides)	17.2		11.4		20.6	

Note: The results are expressed as a percentage of the “LPS” control—the concentration of cytokines in samples that were incubated in the presence of LPS without the addition of peptides; data are presented as medians. *—*p* < 0.05 compared to positive control (LPS), *n* = 4–8 (Mann–Whitney U test). ^#^—*p* < 0.05 compared to negative control (control cells that were incubated under the same conditions in the absence of LPS and peptides), *n* = 4 (Mann–Whitney U test).

## Data Availability

No new data were created.
